# Exploring G Protein-Coupled Receptor Signaling in Primary Pancreatic Islets

**DOI:** 10.1186/s12575-019-0116-y

**Published:** 2020-02-15

**Authors:** Juliane Röthe, Robert Kraft, Torsten Schöneberg, Doreen Thor

**Affiliations:** 1 Rudolf Schönheimer Institute of Biochemistry, Medical Faculty, University of Leipzig, Leipzig, Germany; 2 IFB AdiposityDiseases, University Medical Center, Leipzig, Germany; 3 Carl-Ludwig-Institute for Physiology, Medical Faculty, University of Leipzig, Leipzig, Germany

**Keywords:** GPCR, Second messenger, Primary islets, β cells

## Abstract

**Background:**

Targeting G protein-coupled receptors (GPCRs) in pancreatic cells is feasible to modulate glucose-induced insulin secretion. Because pancreatic islets consist of several cell types and GPCRs can couple to more than one G-protein family, results obtained in pancreatic cell lines do not always match the response in primary cells or intact islets. Therefore, we set out to establish a protocol to analyze second messenger activation in mouse pancreatic islets.

**Results:**

Activation of Gq/11-coupled receptor expressed in primary β cells increased the second messenger IP1 in an accumulation assay. Applying a Gq/11 protein inhibitor completely abolished this signal. Activation of the V1 vasopressin and ghrelin receptors, predominantly expressed in the less abundant alpha and delta cells, was not sufficient to induce a significant IP1 increase in this assay. However, fura-2-based fluorescence imaging showed calcium signals upon application of arginine vasopressin or ghrelin within intact pancreatic islets. Using the here established protocol we were also able to determine changes in intracellular cAMP levels induced by receptors coupling to Gs and Gi/o proteins.

**Conclusions:**

Detection of the second messengers IP1, cAMP, and calcium, can be used to reliably analyze GPCR activation in intact islets.

## Background

Diabetes mellitus is a major metabolic disorder characterized by high glucose levels in the blood which can lead to secondary diseases. Because more than 425 million people world-wide are affected by diabetes, development of novel therapeutics is one of the major current health care challenges. Type 2 diabetes (T2D), accounting for more than 90% of the patients [1], results from insulin resistance of peripheral tissues and impaired β-cell function including altered hormone secretion [2]. The main signal for secretion of insulin, glucagon, and somatostatin is the changing blood glucose level. It is well-established that secretion of all pancreatic islet hormones can be modified by intracellular cyclic AMP (cAMP) and calcium ion levels [3, 4]. G protein-coupled receptors (GPCRs), a superfamily of transmembrane receptors, are the main modulators of these signal molecules. They are considered as major drug targets as they transduce extracellular signals into intracellular responses [5]. Agonists of GPCRs are already used in T2D therapy or are currently in clinical trials [6–9].

Almost 300 GPCRs expressed in pancreatic islets offer multiple opportunities to modulate insulin, glucagon, and somatostatin secretion [10, 11]. However, most of these receptors are still considered orphans because their agonists and/or their signaling properties are currently unknown [12]. Further complexity is added by the fact that most GPCRs couple to multiple G proteins depending on the cell type and cellular environment where they are expressed in [13]. To evaluate the coupling properties of GPCRs in pancreatic islets, islet-derived cell lines are usually analyzed to determine second messenger responses of GPCRs [14–16]. However, results obtained from cell lines suggest aberrant properties, such as a different glucose-induced insulin secretion (GSIS) [17]. Further studies show differences in β cell-derived cell lines and β cells isolated from islets regarding their response to nutrients [18, 19] and their GPCR expression patterns. For example, the β cell-derived cell lines RINm5F and INS-1 express greatly reduced amounts of muscarinic acetylcholine receptor type 3 (M3R) compared to pancreatic islets, and thus, no carbachol (CCh)-induced inositol phosphate accumulation and insulin secretion was detectable in these cell lines [20]. Growth hormone secretagogue receptor/ghrelin receptor (GHS-R) expression has been shown in the β cell-derived cell lines INS-1 and MIN6 [21, 22], while transcriptome analysis of primary islets revealed no GHS-R expression in β cells but only in δ cells [23, 24]. These transcriptome data finally explained the paradox why the Gq/11-coupled GHS-R [25] induced a significant reduction in insulin secretion [26, 27]. The UDP receptor P2Y6 triggered a reduction of GSIS in the β-TC6 cell line while P2Y6 activation in β cells isolated from primary islets increases GSIS [28, 29]. Activation of GPR40 by the agonistic compound GW9508 led to increased GSIS in MIN6 cells but not primary islets [30]. Furthermore, variable coupling properties of GPCRs have been found in different β cell lines. For example, activation of the free fatty acid receptor 2 (FFAR2) by acetate induced a Gq/11 protein-mediated IP1 increase in MIN6 cells but a Gi protein-mediated reduction of cAMP in INS-1 cells [31].

In view of these challenges, methods are required to characterize G protein-signaling cascades in primary pancreatic islets. In particular, the function of a large number of less characterized or orphan GPCRs remains to be clarified in pancreatic islets. Progress was made in monitoring cAMP in primary islets by using genetically modified mouse models expressing FRET-based cAMP sensors [32]. However, this method relies on a transgenic mouse model expressing the cAMP sensor. Therefore, we set out to determine second messenger accumulation in isolated wild-type mouse islets. Thus, we adapted established second messenger protocols routinely performed in cell lines for determining similar responses ex vivo, i.e. in whole or dispersed pancreatic islets. We studied well-characterized GPCRs known to be expressed in pancreatic islets and evaluated incubation procedures as well as islet handling.

## Results

**Fig. 1:**
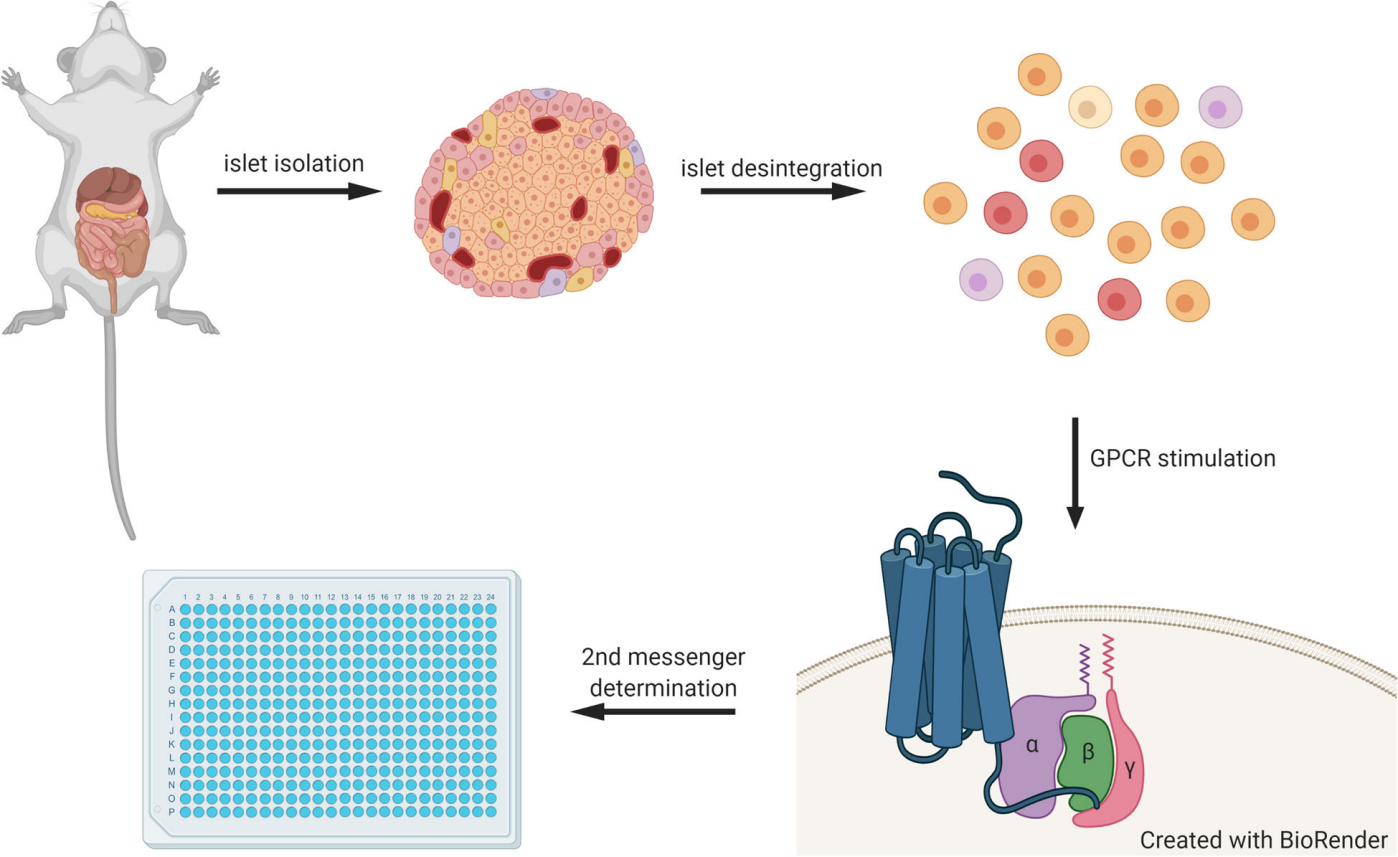
Schematic depiction of the experimental procedures necessary for second messenger analysis in primary islet cells. Islets are isolated by collagenase digestion and disintegrated using Trypsin/EDTA solution. Second messenger determination was carried out in multi-well format

RNAseq-based expression analysis of mouse pancreatic islets [33] revealed highly expressed GPCRs with well-studied signal transduction properties (Table 1). These receptors were used as proof-of-principle to establish protocols for determination of their second messengers. Thus, islets (150 ± 12 per preparation) from mouse pancreata were isolated and disintegrated using trypsin in Ca^2+^-free conditions. After passaging through a 35 μm filter, we obtained 67,000 ± 9000 viable cells per preparation which were sufficient to load 13 wells of a 384-well plate (Fig. 1).

**Table 1:** GPCRs tested in this study. Receptors are given in order from high to low expression [33]

GPCR	Agonist	Coupling properties	Islet expression profile
GLP-1R	GLP-1	Gs	β cell > > α cell > δ cell
SstR1–5	somatostatin	Gi	ubiquitous
P2Y6	UDP	Gq/11	β cell > δ cell > > α cell
M3R	acetylcholine	Gq/11	β cell > δ cell > α cell
FFAR2	acetate	Gi / Gq/11	ubiquitous
V1R	AVP	Gq/11	δ cell = α cell
GHS-R	ghrelin	Gq/11	δ cell > > > α cell > β cell

### IP1 Measurement

**Fig. 2:**
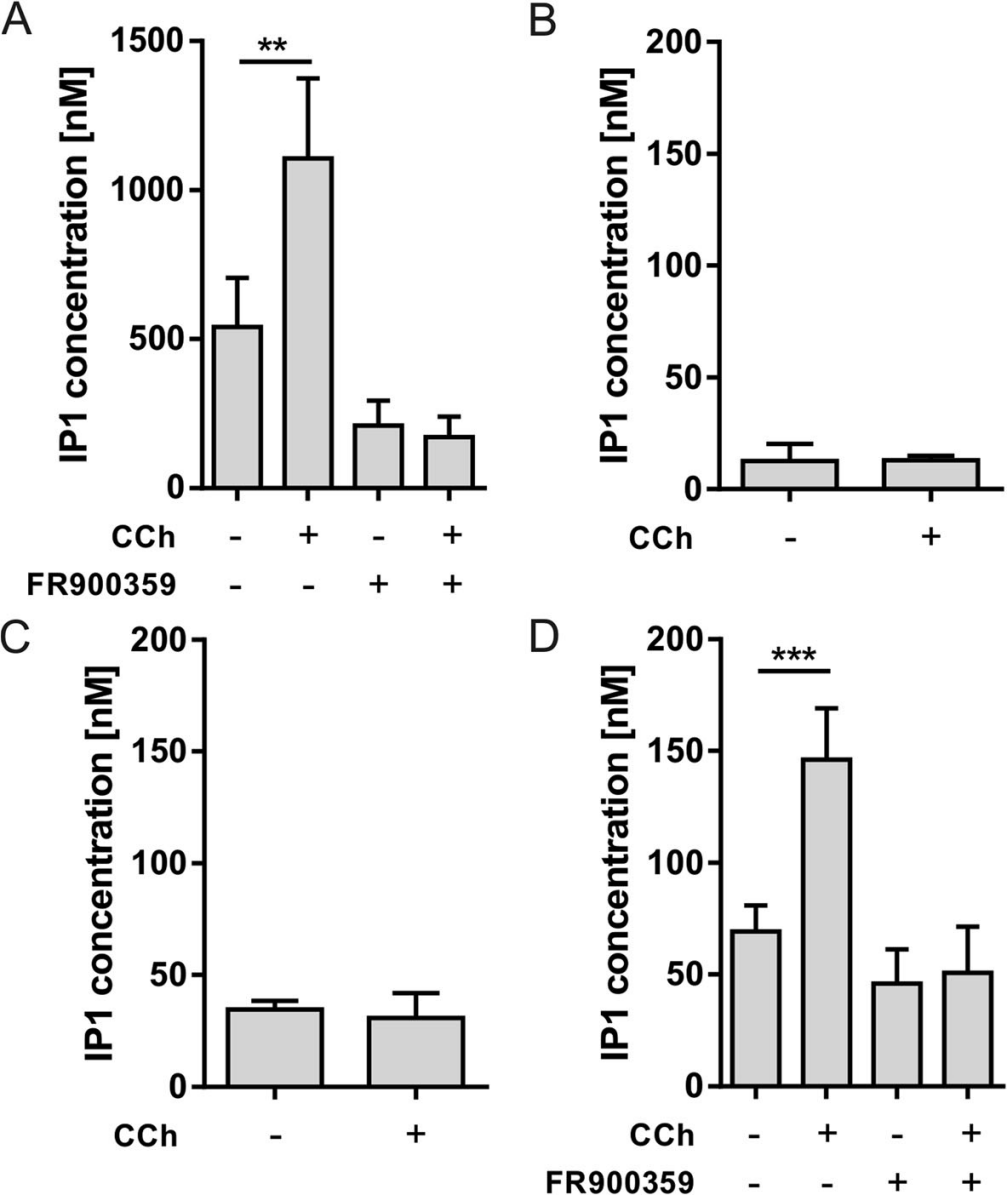
Optimization of conditions to measure IP1 accumulation in dispersed islets. **a** MIN6 cells were seeded into 96-well plates and incubated with 100 μM CCh with or without the Gq/11 inhibitor FR900359. CCh induced a significant increase in IP1 concentration which was completely abolished by FR900359. **b** Dispersed islets were seeded into coated 96-well plates to become adherent overnight and incubated with 100 μM CCh for 4 h, however, CCh failed to induce IP1 accumulation. **c** Using a suspension-based protocol for IP1 determination, dispersed islets were exposed immediately after dispersion to 100 μM CCh for 4 h. CCh also failed to raise IP1 levels in primary islet cells. **d** Reducing the incubation time to 1 h led to a significant increase of IP1 in CCh treated cells. This effect was dependent on Gq/11 activity because application of the Gq/11 inhibitor FR900359 completely abolished IP1 accumulation. Data are given as means ± SEM of three to five independent experiments each performed in triplicates. Statistical significance was tested with the two-tailed paired t test (**p ≤ 0.01; ***p ≤ 0.001)

In a first approach, we used carbachol (CCh) to stimulate M3R, which is highly expressed in the β cell-derived cell line MIN6 and in pancreatic islets, to evaluate optimal conditions for determining the second messenger IP1. For control purposes, MIN6 cells were incubated with CCh to stimulate endogenously expressed receptors and accumulation of IP1 was determined. CCh significantly increased IP1 concentration after 1 h of stimulation to 2.1-fold of the basal level (Fig. 2a). Application of a specific inhibitor for Gq/11 proteins, FR900359 [34], completely abolished basal and CCh-stimulated receptor activity (Fig. 2a). To transfer this established method to primary pancreatic islet cells, dispersed islets were seeded into poly-L-lysine-coated 96-well plates to become adherent overnight. Stimulation with CCh for 4 h at 37 °C did not lead to an increase in IP1 concentration (Fig. 2b). Therefore, an IP1 accumulation protocol for suspension cells was used and CCh was applied for 1 or 4 h. Whereas incubation for 4 h did not lead to an increase in accumulated IP1 (Fig. 2c), stimulation with CCh for 1 h induced a significant increase in IP1 concentration to 210% of the basal level (Fig. 2d). FR900359, the specific inhibitor of Gq/11 proteins completely abolished CCh-induced IP1 accumulation, indicating Gq/11-dependent signal transduction in dispersed islets (Fig. 2d).

**Fig. 3:**
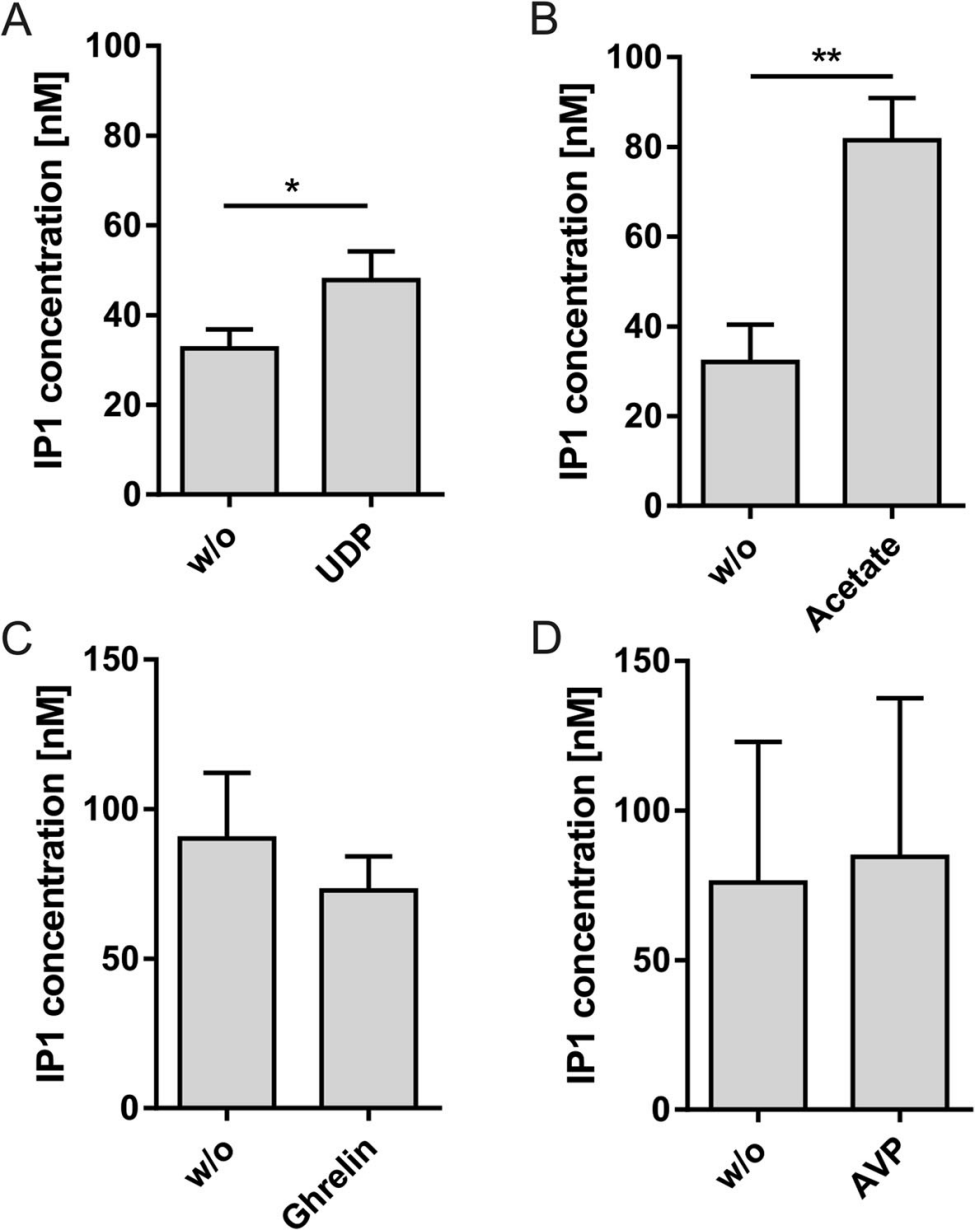
Analyzing IP1 response of different Gq/11 protein-coupled receptors expressed in pancreatic islets. **a**, **b** Stimulation of receptors highly expressed in pancreatic β cells resulted in a significant increase in IP1 concentration. P2Y6 (**a**) was stimulated with 10 μM UDP and FFAR2 (**b**) with 1 mM acetate. **c**, **d** Activation of GHS-R expressed in δ cells by 300 nM ghrelin (**c**) and V1R expressed in α and δ cells by 100 nM AVP (**d**) did not lead to a significant increase of IP1 concentration. Data are given as means ± SEM of three experiments each performed in triplicates. Statistical significance was tested with a two-tailed paired t test (*p ≤ 0.05; **p ≤ 0.01)

We then analyzed IP1 responses of other well-characterized Gq/11-coupled GPCRs (Table 1) displaying high expression in pancreatic islets [33] in suspended cells under the above optimized conditions (Fig. 3). We stimulated disintegrated islets with the P2Y6 receptor agonist UDP (Fig. 3a) and the FFAR2 agonist acetate (Fig. 3b) and observed a significant increase in IP1 concentration by 1.5- and 2.5-fold, respectively. This data indicates that activation of receptors highly expressed in pancreatic β cells induce a measurable IP1 accumulation. However, stimulation of receptors predominantly expressed in α and δ cells was not sufficient to induce a significant IP1 response. Neither activation of growth hormone secretagogue receptor/ghrelin receptor (GHS-R) in δ cells (Fig. 3c) nor stimulation of vasopressin receptor type 1 (V1R) by arginine vasopressin (AVP) in α and δ cells (Fig. 3d) resulted in an increase of intracellular IP1 concentration.

### cAMP Measurement

**Fig. 4:**
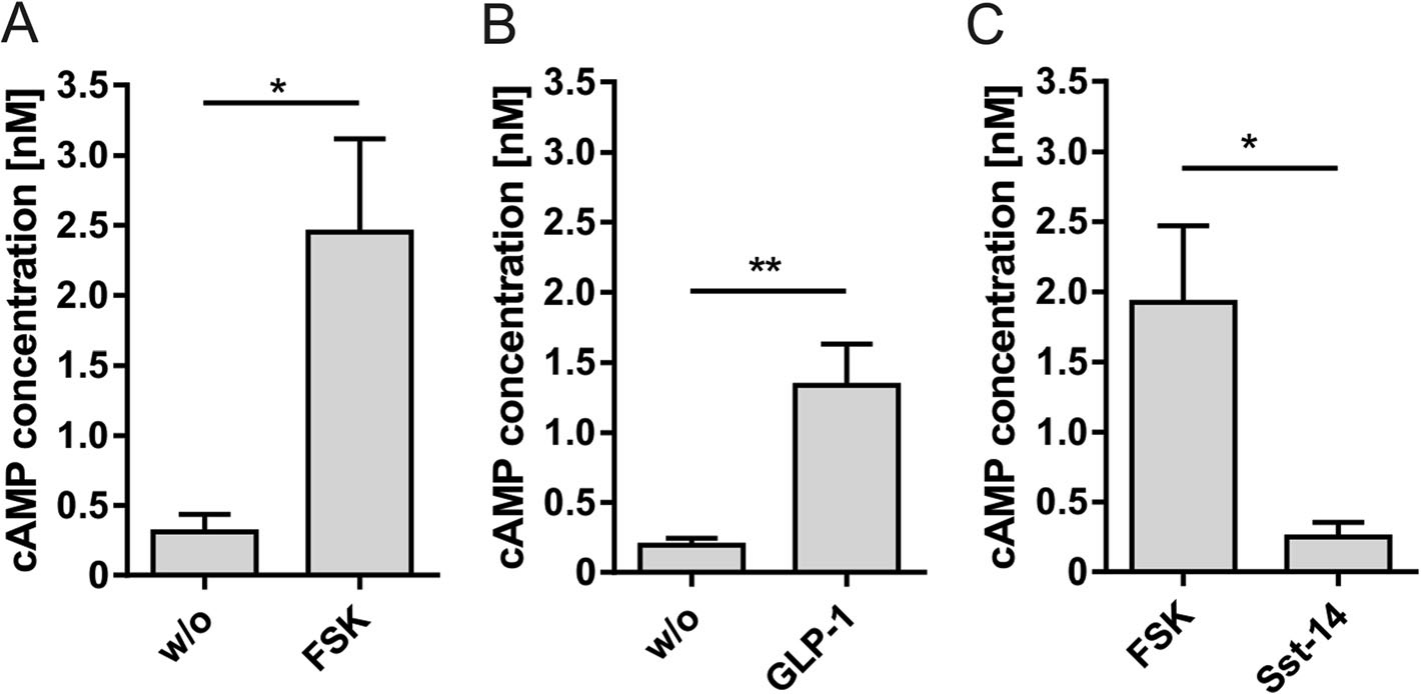
Analyzing cAMP levels in response to activation of Gs- or Gi/o protein-coupled receptors in dispersed islets. **a** The unspecific activation of adenylyl cyclases by 10 μM forskolin resulted in an almost 8-fold increase of cAMP. **b** Activation of the highly expressed GLP-1R by its agonist induced a significant increase in cAMP (> 6-fold). **c** Incubation with 1 μM forskolin increased intracellular cAMP concentration which can be reduced by 1 μM Sst-14 due to activation of Gi/o protein-coupled SstR. Given are the means ± SEM of four to five experiments each performed in triplicates. Statistical significance was tested with the two-tailed paired t test (*p ≤ 0.05; **p ≤ 0.01)

Activation of GPCRs coupling to either Gs- or Gi/o proteins changes the intracellular cAMP concentration which in turn modulates pancreatic hormone secretion. To optimize conditions for intracellular cAMP determination we initially stimulated adenylyl cyclases nonspecifically with forskolin (Fig. 4a). The most stable cAMP accumulation was achieved by simultaneous incubation of islet cells with forskolin and the beads necessary for cAMP detection. We next targeted glucagon-like peptide 1 receptor (GLP-1R), the most abundant GPCR in pancreatic islets [33]. Incubation of pancreatic islet cells with 100 nM GLP-1 yielded a significant increase of cAMP concentration to 690% of the basal level (Fig. 4b). For analysis of Gi/o protein-coupled receptors, a preceding stimulation with forskolin is required to achieve reducible cAMP concentrations. We stimulated ubiquitously expressed somatostatin receptors (SstR) with somatostatin (Sst-14) and observed a potent reduction of the forskolin-induced cAMP levels to 13% compared to forskolin-stimulated islet cells (Fig. 4c).

### Ca^2+^ Imaging of Pancreatic Islets

**Fig. 5:**
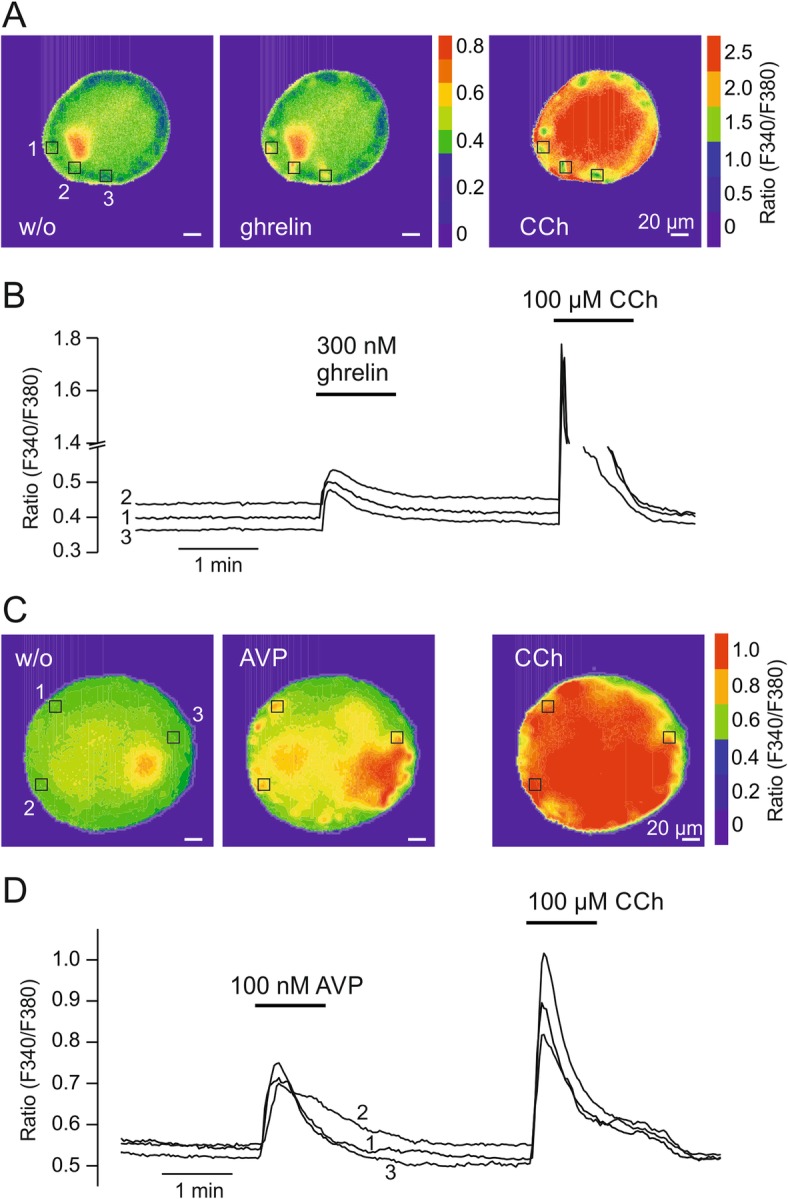
Ca^2+^ imaging of pancreatic islets. **a** Ca^2+^ imaging experiments were performed in single pancreatic islets loaded with fura-2 AM. Images show the fluorescence ratio (F340/F380) of one islet before and after addition of 300 nM ghrelin and 100 μM CCh. **b** Time course of Ca^2+^ responses in three regions within the islet is shown and corresponds to (**a**). **c** Images show the fluorescence ratio (F340/F380) of another islet before and after addition of 100 nM AVP and 100 μM CCh. **d** Time course of Ca^2+^ responses in three regions within the islet is shown and corresponds to (**c**)

Measurement of the second messengers cAMP and IP1 seems feasible for receptors which couple to Gq/11, Gs, or Gi proteins and show expression in the abundant pancreatic β cells. Because the Gq/11 protein-coupled V1R and GHS-R are mainly expressed in α and δ cells, we tested the ability of AVP and ghrelin, respectively, to induce Ca^2+^ signals in these less abundant islet cell types. Ca^2+^ imaging of dispersed primary islet cells and intact pancreatic islets using the fluorescent dye fura-2 has been previously described [35, 36]. One disadvantage of Ca^2+^ measurement in intact islets is the slow adhering process to coverslips which lasts for several days [36]. Therefore, we coated glass coverslips with poly-L-lysine and observed reliable adherence of islets within 48 h enabling perfusion of extracellular solutions. To induce Ca^2+^ signals we loaded intact islets with fura-2 AM and applied the GHS-R agonist ghrelin or the V1R agonist AVP via the bath solution. For control purposes, CCh was perfused subsequently to activate Gq/11 protein-coupled M3R which are mainly expressed in β cells. Ghrelin induced a detectable Ca^2+^ signal in some regions of the islet whereas CCh evoked a large response in all parts of the islet (Fig. 5a, b). Application of AVP evoked robust Ca^2+^ elevations in several regions of the islet and CCh again induced a widespread Ca^2+^ signal (Fig. 5c, d). These results indicate the functionality of both receptors in pancreatic islets.

### Second Messenger Analysis in Islets Displaying Reduced Insulin Secretion

**Fig. 6:**
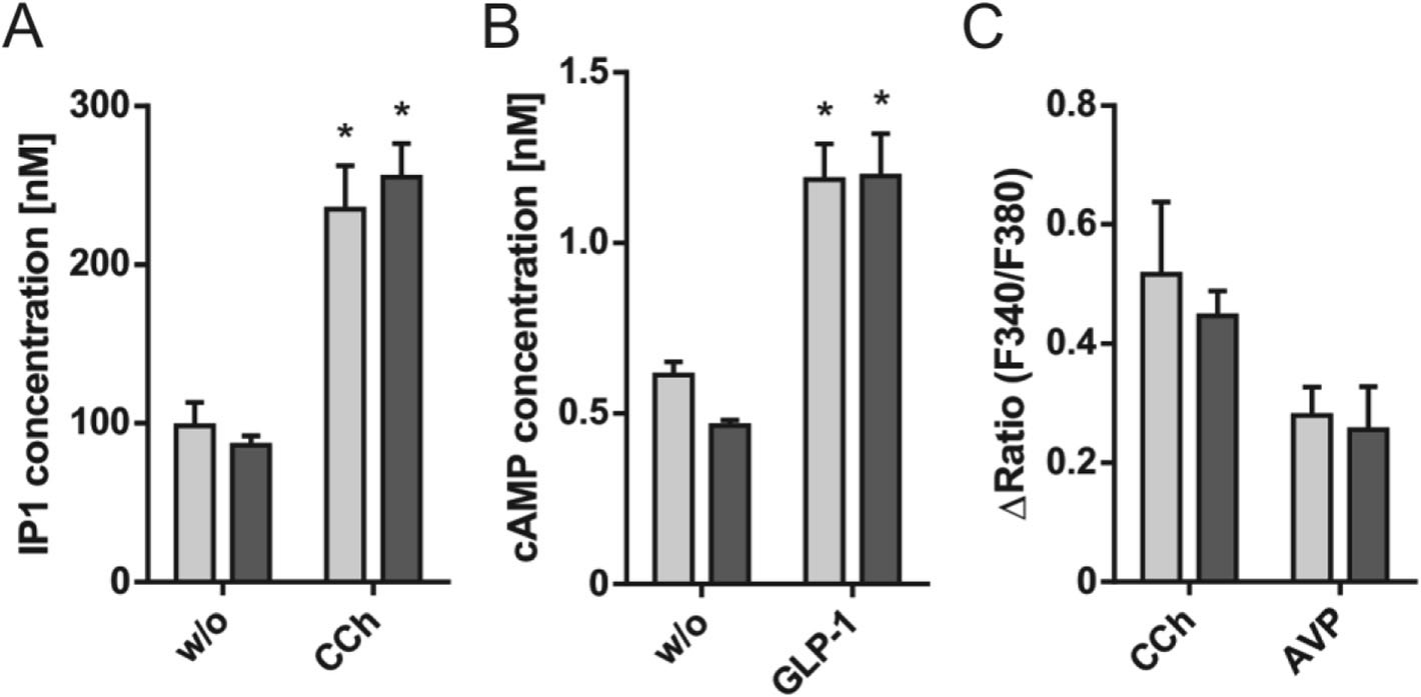
Determining second messenger responses in islets of a P2Y14-deficient mouse model. **a** Activation of the Gq protein signaling pathway by the muscarinic receptor agonist CCh (100 μM) induces significant increases in IP1 concentration in wild-type (light grey) and P2Y14 knock-out (dark grey) islets. **b** Activation of GLP-1R using 100 nM GLP-1 induced a significant increase in cAMP concentration in wild-type and P2Y14 knock-out islets. Shown are the means ± SEM of two independent experiments each performed in duplicates. Statistical significance (w/o vs. stimulus) was tested with the two-tailed unpaired t test (*p ≤ 0.05). **c** Ca^2+^ imaging experiments were performed in the presence of CCh (100 μM) and AVP (100 nM) in single pancreatic islets from wild-type and P2Y14 knock-out mice loaded with fura-2 AM. Stimulus-evoked changes in the fluorescence ratio (F340/F380) were obtained by subtracting basal from peak value of 10 regions within each islet. Mean values of ΔRatio (F340/F380) ± SEM were calculated from mean values of regions of each wild-type and P2Y14 knockout islet (*n* = 3)

T2D is not only characterized by insulin insensitivity of the peripheral tissue but also by inadequate insulin secretion upon glucose treatment which finally results in an impaired glucose tolerance. Increasing insulin secretion by modulating second messenger response is one strategy to improve glucose tolerance [6]. Therefore, an evaluation of the second messenger response in dysfunctional pancreatic islets can help to identify receptor targets to increase insulin secretion. We, therefore, used a P2Y14 knock-out mouse model with reduced glucose-induced insulin release [33] to assess islet responsiveness to GPCR agonists. Islets from knock-out animals and wild-type littermates were stimulated with the previously tested agonists CCh for IP1 measurements, GLP-1 for cAMP determination, as well as CCh and AVP to assess Ca^2+^ signals. All tested substances induced a significant increase in second messenger concentrations. However, neither IP1, cAMP, nor Ca^2+^ signals differed between wild-type and knock-out mice (Fig. 6a-c), suggesting that second messenger responsiveness in knock-out islets is not impaired and that the reduced glucose-responsiveness does not depend on changed second messenger amounts.

## Discussion

In pancreatic islets, extracellular signals modulating hormone secretion are often mediated by GPCRs and subsequent changes of intracellular second messengers [37]. Because GPCRs may represent novel targets in diabetes therapy [38, 39], evaluating the impact of receptor activation is necessary. Several cell lines representing different pancreatic cell types [15] have been used to study the effect of GPCR activation.

M3R is a well-characterized GPCR with numerous agonists, inverse agonists and allosteric modulators. Pharmacological studies showed that M3R activation results in generation of inositol (1,4,5)-trisphosphate via coupling to Gq/11 proteins [40]. As MIN6 cells have been shown to functionally express M3R [41], we used this cell line to measure a CCh-induced IP1 signal. As expected, receptor activation by CCh raised intracellular IP1 by approximately 2-fold, an effect depending on Gq/11-protein activity (Fig. 2a). Applying the same protocol, we determined IP1 accumulation in dispersed islets, but no signal was detectable in adherent islet cells most probably due to the low number of cells attached in 96-well plates. Using dispersed islets directly to determine agonist-induced IP1 accumulation we observed a 2-fold increase upon CCh application similar to the response in MIN6 cells (Fig. 2a, d). In addition, CCh induced a strong Ca^2+^ signal in pancreatic islets (Fig. 5) as reported previously [41].

The same protocol was applied to other Gq/11 protein-coupled receptors expressed in pancreatic islets [33]. Of the tested agonists, UDP and acetate were able to induce a significant increase in IP1 accumulation in primary islet cells by about 1.5-fold and 2.5-fold, respectively. Previous results reporting UDP stimulation of P2Y6 are contradicting. While UDP reduced GSIS in β-TC6 cells [28], other groups observed an increased GSIS in β-TC6 cells and primary islets induced by UDP [29, 42]. The latter reports are in line with pharmacological characterization of P2Y6 using UDP stimulation [43] and our data showing an increased IP1 concentration, indicating Gq/11 protein activation. Acetate has been shown to induce a Gq/11 protein-mediated Ca^2+^ elevation in the pancreatic β cell line β-TC3 [44] and to increase IP1 concentration in MIN6 cells and primary mouse islets by about 1.5-fold [31], indicating that the here described method provides robust signals.

We could not detect IP1 signals for GHS-R or V1R in pancreatic islets stimulated with ghrelin and AVP, respectively. Recent RNAseq analysis data revealed the repertoire of GPCRs specifically expressed in the different pancreatic islet cell types [23, 24]. GHS-R and V1R are predominantly expressed in α and/or δ cells [23, 24], cell types with a markedly lower abundancy (about 10–15% and 5–10% of total cell number, respectively) in pancreatic islets [45]. It can, therefore, be assumed that the number of cells expressing these receptors limits signal detection. However, for Gq/11 and Gi/o protein-coupled receptors, imaging of intracellular Ca^2+^ with a resolution suitable for single cells might be one strategy to circumvent this problem. As showed for GHS-R, ghrelin-induced receptor activation is detectable by an increase of intracellular Ca^2+^ within regions of whole pancreatic islets (Fig. 5a). Although previous work showed effects of ghrelin on HIT-T15 and INS-1E β cells [46, 47], recent work demonstrated a cell-type restricted expression of GHS-R to pancreatic δ cells [23, 24] which is compatible with our Ca^2+^ imaging experiments. AVP has been shown to increase intracellular Ca^2+^ in In-R1-G9, an α cell-derived cell line, resulting in secretion of glucagon [48, 49]. V1R expression has also been claimed in pancreatic β cells with insulin secretion upon AVP stimulation being dependent on phospholipase C and adenylyl cyclase [49–51]. However, current transcriptome data does not support expression of AVP receptors in pancreatic β cells [23, 24]. Here, we show that similarly to ghrelin AVP does not induce a measurable increase in intracellular IP1 concentration but receptor activation can be monitored by Ca^2+^ imaging experiments within regions of the islet (Fig. 5c).

We finally applied the protocol established for islet dispersion for measurement of receptor-stimulated cAMP accumulation. Determination of cAMP in pancreatic islets has been performed previously using a transgenic mouse model containing a cAMP reporter [52]. This model allows for imaging of cAMP fluctuations. However, it requires tetracycline transactivator to achieve cell-type specific activation which is more time- and resource-consuming. The here established protocol can be used for wild-type islets as well as for islets isolated from diverse knock-out mouse models to evaluate second messenger responses without requiring cross-breeding with reporter mice. Another approach used isolated islets to determine cAMP upon glucose administration [53], however, this protocol recommends the usage of typically 25 to 50 islets per sample for optimal cAMP determination. Because one preparation typically yields 150 islets per mouse, it is insufficient to analyze different receptors. We have used forskolin, GLP-1, and SSt to evaluate changes in cAMP (Fig. 4). Forskolin, an unspecific activator of adenylyl cyclases, and the incretin GLP-1 significantly raised intracellular cAMP by 8-fold and 6.5-fold, respectively. GLP-1R activation has been previously shown to increase cAMP concentration in the β cell-derived cell lines INS-1E and MIN6 by about 3- and 2-fold, respectively [54, 55]. Somatostatin was shown to inhibit the cAMP response in INS-1 cells pretreated with 1 μM forskolin [56]. We found a clear reduction of forskolin-induced cAMP accumulation by 87%. Thus, our data demonstrate that the here presented protocol for measurement of cAMP in primary islets shows adequate sensitivity, is resource-sparing, and does not require transgenic cAMP reporter mice.

Because modulating second messenger responses is one option to overcome reduced insulin secretion, we finally evaluated this protocol on pancreatic islets with impaired function [33]. Islets from P2Y14 knock-out animals display stimulus-dependent increases in IP1 and cAMP concentrations as well as intracellular Ca^2+^ signals. These responses are comparable to those induced in wild-type islets suggesting that the impaired insulin secretion is not a result of an overall changed second messenger responsiveness in this mouse model. These data further indicate that our method can be used to evaluate the effect of receptor activation also in mouse models of pancreatic islet dysfunction. Since comparable islet cell number and viability is essential for the here described method, models of islet dysfunction induced by streptozotocin treatment might not be suitable for our analysis because streptozotocin drastically reduces islet cell number [57].

Furthermore, the control of cell vitality within individual preparations is necessary to obtain reliable results. We, therefore, suggest stimuli like CCh and forskolin, which induce strong and highly reproducible responses as control compounds to eliminate false-negative results.

## Conclusion

With the ongoing discussion about the suitability of pancreatic cell lines for studying functions of pancreatic islets [58, 59], the usage of primary islets can help to clarify contradictory results. Here, we present optimized protocols to determine receptor-mediated second messenger levels of IP1 and cAMP in primary islets. Compared with existing protocols using transgenic reporter animals, islets obtained from wild-type animals are sufficient for the here described methods. Further, due to improved sensitivity, less islet cells are required and, therefore, several different conditions can be tested from one preparation.

## Methods

### Materials

If not mentioned otherwise, reagents and standard chemicals purchased from Sigma-Aldrich or Carl Roth. Cell culture media and reagents were obtained from Gibco (ThermoFisher Scientific) and poly-L-lysine (MW > 300,000) from Biochrom. Cell culture material was purchased from Sarstedt.

### Cell Culture

MIN6 cells were grown in Dulbecco’s minimum essential medium supplemented with 15% fetal bovine serum (FBS), 100 U/ml penicillin, 100 μg/ml streptomycin, and 142 μM mercaptoethanol in a humidified incubator with 5% CO_2_/ 95% air at 37 °C.

IP1 measurements were performed using the IP-One Tb kit (Cisbio). Thus, 50,000 MIN6 cells were seeded per well of a 96-well plate. Two days after seeding, cells were washed with stimulation buffer containing 10 mM LiCl and incubated with 100 μM CCh for 60 min. Cells were lysed using the provided lysis buffer and IP1 levels were determined (see below).

### Animals

C57BL6/N mice were bred under specific pathogen-free conditions on 12 h light/ 12 h dark cycle, 21 °C, and 55% humidity with ad libitum access to food and water. All experiments were conducted in accordance with European Directive 2010/63/EU on the protection of animals used for scientific purposes and were performed with permission from the Animal Care and Use Committee (ACUC# T24/16; ACUC# T19/18) and the Government of the State of Saxony, Germany. Mice were between 10 and 14 weeks of age and were matched to age and gender.

### Isolation and Culture of Murine Islets

Pancreatic islets were isolated from mice sacrificed by cervical dislocation. Collagenase P was dissolved (0.5 mg/ml) in ice-cold Krebs-Ringer buffer (KRB: 115 mM NaCl, 4.7 mM KCl, 1.2 mM KH_2_PO_4_, 1.2 mM MgSO_4_, 2.56 mM CaCl_2_, 20 mM NaHCO_3_, 10 mM Hepes, 0.1% BSA, 5 mM glucose, pH 7.3) and was injected via the common bile duct into pancreas. Afterwards, the distended pancreas was digested in shaking water bath at 37 °C for 12–15 min. Vital islets were washed twice in warm KRB and hand-picked under the stereomicroscope. Isolated islets were cultured in RPMI containing 10% FBS, 100 U/ml penicillin, and 100 μg/ml streptomycin overnight at 37 °C and 5% CO_2_/ 95% air in a humidified atmosphere.

### Islet Dispersion

All reagents were warmed in a 37 °C water bath. Islets were washed in Ca^2+^-free KRB (115 mM NaCl, 4.7 mM KCl, 1.2 mM KH_2_PO_4_, 1.2 mM MgSO_4_, 20 mM NaHCO_3_, 10 mM Hepes, 0.1% BSA, 5 mM glucose, pH 7.3) containing 1 mM EGTA and transferred into a petri dish. Vital islets were hand-picked into a 1.5 ml reagent tube and centrifuged at 200 rpm for 1 min. The buffer was removed and islets were resuspended in 200 μl Ca^2+^-free KRB containing 1 mM EGTA and 0.05% Trypsin/EDTA by gently pipetting. After 60 s of incubation, 1 ml RPMI media with 10% FBS was carefully added. After centrifugation (1 min, 2000 rpm) islets were suspended in 500 μl RPMI media and transferred through a 35 μm filter (Falcon). After washing with 500 μl RPMI media, cells were transferred to the respective assay buffer. Cell number was determined by counting using a Neubauer chamber.

### IP1 Accumulation Measurement

For analysis of adherent islet cells, 10,000 cells/well were seeded into poly-L-lysine-coated 96-wells plates (0.1 mg poly-L-lysine/ml) to become adherent overnight. Stimulation was performed in 35 μl of assay buffer (Cisbio) containing 10 mM LiCl and the indicated agonists. After 60 min incubation at 37 °C in a humidified incubator, cells were lysed with 30 μl lysis buffer provided by the manufacturer. Lysats were kept frozen until IP1 measurement using the IP-One Tb kit (Cisbio). Thus, 14 μl of lysed stimulated cells were transferred to ProxiPlate-384 Plus microplates (PerkinElmer Life Sciences) and incubated with 3 μl acceptor-bead solution and 3 μl donor-bead solution (bead stock solutions were diluted 1:20 in lysis buffer). After 60 min incubation at room temperature in the dark, fluorescence was determined using the EnVision Multilabel Reader (PerkinElmer Life Sciences) by excitation at 330 nm and emission at 620 nm.

To analyze IP1 accumulation in suspension cells, 5000 islet cells/well were incubated in ProxiPlate-384 Plus microplates (PerkinElmer Life Sciences) with the indicated substances to stimulate GPCR in 14 μl assay buffer (Cisbio) containing 10 mM LiCl. After the indicated time points, 3 μl acceptor-bead solution and 3 μl donor-bead solution (1:20 dilution in lysis buffer) were added. After 60 min incubation at room temperature in the dark, fluorescence was determined using the EnVision Multilabel Reader (PerkinElmer Life Sciences) by excitation at 330 nm and emission at 620 nm.

### cAMP Accumulation Measurement

The cAMP accumulation was determined in 384-well white OptiPlate microplates (PerkinElmer Life Sciences) using the AlphaScreen cAMP assay kit (PerkinElmer Life Sciences). Thereto, dispersed islets (5000 cells/well) were resuspended in cAMP stimulation buffer (HBSS containing 0.5 mM IBMX, 5 mM Hepes, 0.1% BSA, pH 7.4) with the indicated substances in a final volume of 10 μl containing 0.1 μl of cAMP acceptor beads. After 30 min 15 μl of donor-bead solution containing 0.1 μl streptavidin donor beads and 0.3 μl biotinylated cAMP in lysis buffer (5 mM Hepes, 0.1% BSA, 0.3% Tween-20, pH 7.4) was added and incubated for further 60 min at room temperature. Values were determined by excitation at 640 nm and emission at 570 nm using the EnVision Multilabel Reader (PerkinElmer Life Sciences).

### Ca^2+^ Imaging

Pancreatic mouse islets were transferred into 12-well plates 1 day after preparation on poly-L-lysine coated glass coverslips (12 mm diameter, 3–4 islets per coverslip). Two days after seeding, coverslips were transferred into a new 12-well plate, where islets were loaded with 5 μM fura-2 AM dissolved in standard bath solution for 60 min at 20–22 °C. The standard bath solution contained 140 mM NaCl, 10 mM Hepes, 5 mM KCl, 2 mM CaCl_2_, 1 mM MgCl_2_, and 3 mM glucose (pH 7.4). After loading, the coverslip was transferred to a perfusion chamber (Warner Instruments) mounted on an upright microscope (Olympus BX51WI). Islets were visualized using a 20x water immersion objective (UMPlanFl, Olympus) and perfused with standard bath solution and stimulated by adding 300 nM ghrelin, 100 nM AVP, or 100 μM CCh to standard bath solution. Fura-2-based Ca^2+^ imaging was performed in intact pancreatic islets using a monochromator-based imaging system and the imaging software TILLvisION 4.0 (both T.I.L.L. Photonics). Emitted fluorescence at 510 nm (excited at 340 nm and 380 nm) was collected with a CCD camera (PCO Imaging), acquired at intervals of 2 s and corrected for background fluorescence.

### Statistical Analysis

If not stated otherwise, data is presented as means ± SEM. Statistical analysis was performed using GraphPad Prism version 6.0. As stated in the figure legends, data was analyzed using a two-tailed paired student’s t test. *P*-values with *p* ≤ 0.05 were considered statistically significant.

## Data Availability

Data used and analyzed in this study are included in this article. Transcriptome data were performed by [33] and GPCR expression analyzed from the given FPKM value.

## Abbreviations

AVP: arginine vasopressin
cAMP: 3′,5′-cyclic adenosine monophosphate
CCh: carbachol
FFAR2: Free fatty acid receptor type 2
GHS-R: growth hormone secretagogue receptor/ghrelin receptor
GLP-1R: glucagon-like peptide-1 receptor
GPCR: G protein-coupled receptor
GSIS: glucose-induced insulin secretion
M3R: muscarinic acetylcholine receptor type 3
Sst-R: somatostatin receptor
T2D: type 2 diabetes
UDP: uridine 5′-diphosphate
